# A Review on a Hidden Gem: Phycoerythrin from Blue-Green Algae

**DOI:** 10.3390/md21010028

**Published:** 2022-12-29

**Authors:** Hui Teng Tan, Fatimah Md. Yusoff, Yam Sim Khaw, Nur Amirah Izyan Noor Mazli, Muhammad Farhan Nazarudin, Noor Azmi Shaharuddin, Tomoyo Katayama, Siti Aqlima Ahmad

**Affiliations:** 1 Laboratory of Aquatic Animal Health and Therapeutics, Institute of Bioscience, Universiti Putra Malaysia, Serdang 43400, Selangor, Malaysia; 2 Department of Aquaculture, Faculty of Agriculture, Universiti Putra Malaysia, Serdang 43400, Selangor, Malaysia; 3 International Institute of Aquaculture and Aquatic Sciences, Universiti Putra Malaysia, Port Dickson 71050, Negeri Sembilan, Malaysia; 4 Department of Biochemistry, Faculty of Biotechnology and Biomolecular Sciences, Universiti Putra Malaysia, Serdang 43400, Selangor, Malaysia; 5 Graduate School of Agricultural and Life Sciences, The University of Tokyo, 1-1-1, Yayoi, Bunkyo, Tokyo 113-8657, Japan

**Keywords:** cyanobacteria, extraction, purification, application, stability, commercial, cultivation

## Abstract

Phycoerythrin (PE) is a pink/red-colored pigment found in rhodophytes, cryptophytes, and blue-green algae (cyanobacteria). The interest in PE is emerging from its role in delivering health benefits. Unfortunately, the current cyanobacterial-PE (C-PE) knowledge is still in the infant stage. It is essential to acquire a more comprehensive understanding of C-PE. This study aimed to review the C-PE structure, up and downstream processes of C-PE, application of C-PE, and strategies to enhance its stability and market value. In addition, this study also presented a strengths, weaknesses, opportunities, and threats (SWOT) analysis on C-PE. Cyanobacteria appeared to be the more promising PE producers compared to rhodophytes, cryptophytes, and macroalgae. Green/blue light is preferred to accumulate higher PE content in cyanobacteria. Currently, the prominent C-PE extraction method is repeated freezing–thawing. A combination of precipitation and chromatography approaches is proposed to obtain greater purity of C-PE. C-PE has been widely exploited in various fields, such as nutraceuticals, pharmaceuticals, therapeutics, cosmetics, biotechnology, food, and feed, owing to its bioactivities and fluorescent properties. This review provides insight into the state-of-art nature of C-PE and advances a step further in commercializing this prospective pigment.

## 1. Introduction

Phycoerythrin (PE) is a colored, water-soluble pigment–protein complex from the phycobiliprotein (PBP) family and is predominantly found in rhodophytes, cryptophytes, and cyanobacteria [1,2]. It is a 240 kDa oligomeric chromoprotein with an intense pink/red color [3]. This PBP is a light-harvesting accessory pigment that absorbs wavelengths that chlorophyll cannot [4]. In terms of energy transfer, PE is the first PBP to absorb light and emits energy to phycocyanin (PC) and allophycocyanin (APC), consecutively [5]. PE is characterized by its original spectrum properties, such as fluorescence emission [6]. On the other hand, previous PBP research was focused on PC, and it was only recently that the scientific community realized that PE is endowed with antioxidant potential due to its ability to scavenge free radicals [7,8,9]. This allows them to prevent or delay the onset of potentially fatal diseases, such as cancer, heart disease, and hepatotoxicity. The potent antioxidant activity promotes human health and boosts the immune response against infections [10]. These bioactive compounds also resist photooxidative damage, which would otherwise have an adverse impact on crucial cellular machineries, such as proteins, lipids, and DNA [11,12]. In comparison to PC and APC, the unique fluorescent properties of PE have made it a useful tool in the diagnosis and treatment of tumors [13]. PE has gained prominence not only as pharmaceuticals, nutraceuticals, cosmetics, and colorants but also as biologically active compounds, which will be described in this review.

PE is generally classified into three types based on different spectral properties and the photosynthetic organisms that produce them—namely, B-phycoerythrin (B-PE), C-phycoerythrin (C-PE), and R-phycoerythrin (R-PE) from Bangiophyceae (primitive filamentous Rhodophyta), cyanobacteria, and complex Rhodophyta, respectively [14,15]. B-PE and R-PE from red algae have been well-studied and widespread in their use across various industries [13,16]. B-PE became a valuable candidate as an analytical reagent and in fluorescent technologies, owing to its large absorption coefficient and high fluorescence efficiency (high quantum yield and high stokes shift) [14]. In contrast, C-PE derived from cyanobacteria received relatively little attention due to public concern about cyanotoxins.

In recent years, previous reviews have reported on the scientific progress and challenges of microalgal pigments [17,18,19]. Instead of a detailed report on C-PE, the existing publications focused on the general characteristics and features of cyanobacterial PBP, particularly C-PC, while overlooking the specific strains, characteristics, and upstream and downstream processes specifically optimized for C-PE as well as the specific applications of C-PE [20,21]. *Spirulina platensis* is the most commonly used strain since it has been recognized as an effective multiple-product-producing strain for decades [20]. Nevertheless, there is a growing need to explore the cyanobacterial world in order to discover new potential strains for C-PE production. Furthermore, there is a dearth of exclusive reports on C-PE, maximum C-PE valorization, and the possibility of bolstering novel strains for C-PE production. Hence, this review discusses C-PE and its various benefits and applications. It also presents an abridged overview of a decade-long journey by highlighting research areas from scientific literature published on C-PE production. This review also featured a SWOT (strengths, weaknesses, opportunities, and threats) analysis to determine the internal and external factors and obstacles associated with C-PE commercialization.

### The Odyssey of Phycoerythrin Research

The necessity of developing naturally derived bioactive compounds capable of preventing diseases motivated researchers to explore various algae or plant sources in pursuit of novel and sustainable pigment supplies. This sector experienced massive development in the 1970s when public awareness shifted toward health safety and a predilection for naturally derived pigments over chemically synthesized pigments [22]. This has resulted in a growing interest in studying cyanobacteria as a potential source due to the possibilities of commercial applications in several fields. More specifically, PBP extracted from cyanobacteria has been studied and reported to have anticancer, antioxidant, anti-inflammatory, and antimicrobial properties, making it a priority for industrial production in various lucrative sectors, such as pharmaceuticals, foods and feeds, cosmetics, and nutraceuticals [16]. Aside from the well-known PC, PE, with its excellent fluorescent features, has progressively become a research focus. The advent of PE as a fluorescent marker for macromolecules and cells was followed by its widespread use in high-sensitivity fluorescence technologies [14]. Most PE sources are limited to red algae, especially *Porphyridium* sp., which have long been studied and commercially exploited for R-PE [23,24]. However, cyanobacteria can become an alternative source. C-PE may enter the mainstream due to their fast and easy cultivation, complementary chromatic adaptation (CCA) ability, and greater resistance to bacterium contamination [3,25].

## 2. Structure of C-PE 

**Figure 1:**
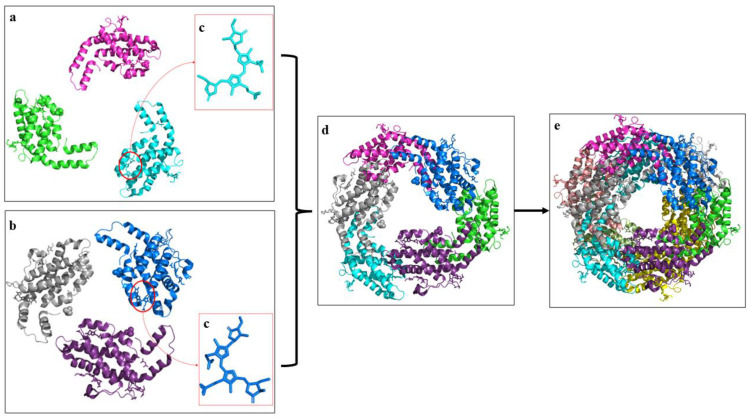
Atomic resolution structure (PDB ID: 5NB4) of C-phycoerythrin from *Phormidium* sp. (**a**) α-subunits; (**b**) β-subunits; (**c**) three-dimensional structure of phycoerythrobilin; (**d**) three-dimensional structure of trimer; (**e**) three-dimensional structure of hexamer (adopted from [33] with permission, 2017, Sonani et al.).

**Figure 2:**
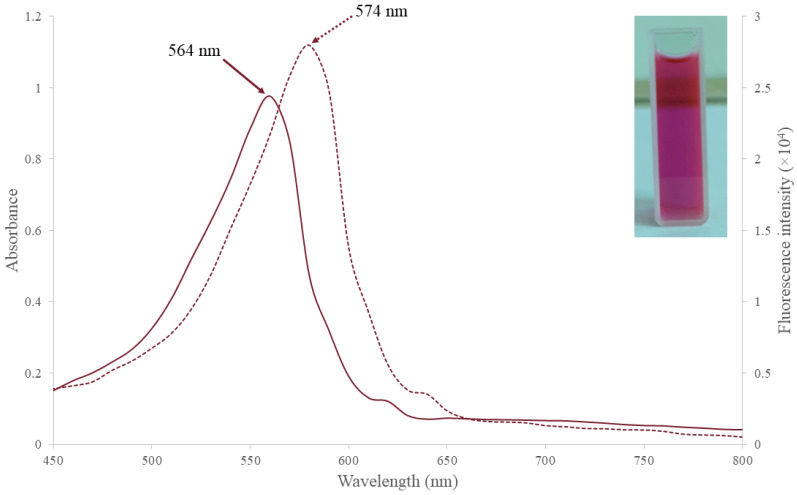
The UV-visible absorbance spectra (solid line) and fluorescence emission spectra (dotted line) of C-PE extracted from *Pseudanabaena* sp. (UPMC-A0103). C-PE of *Pseudanabaena* sp. showed an absorption maxima at 564 nm and fluorescence emission maxima at 574 nm.

Each PE comprises two different polypeptides (denoted as α and β), with each subunit containing a protein backbone and a phycobilin bound by a covalent bond (Figure 1) [16]. The *cpe*A gene encodes the α subunit of PE, while the *cpe*B gene encodes the β subunit [26]. The three types of PE (C-PE, R-PE, and B-PE) differ in the number and type of chromophores attached, resulting in different absorption maxima. Unlike R-PE and B-PE, C-PE has only one absorption maximum, at about 564 nm (Figure 2) [27]. C-PE is produced mainly by cyanobacteria and is further subdivided into C-PE-I and C-PE-II [28]. There are four forms of phycobilins in cyanobacteria, each with a different color: phycocyanobilin (PCB) in blue, phycourobilin (PUB) in yellow, phycoerythroblin (PEB) in red, and phycoviolobilin (PVB) in violet [29]. These phycobilins have been reported to be one of the major components responsible for the bioactivity of PE [16]. Each form of phycobilin has a system of conjugated double bonds that give it a unique spectral characteristic that can be further modified by interactions between the subunits and the chromophore [30]. Marine and freshwater or soil cyanobacteria have different phycobilin contents. PE from freshwater and soil cyanobacteria carry PEB chromophores solely, with absorption and fluorescence emission spectra resembling the spectral properties of C-PE-I [31]. C-PE-I carried five PEBs (αβ) that were covalently bonded to the cysteine (Cys) residue (α-84, α-140 or β-143, β-84 or β-82, β-50 or β-61, and β-155 or β-159) [21]. C-PE-I is a disk-shaped hexamer formed by two trimeric (αβ)_3_ through face-to-face aggregation. It is typically found in the form of (αβ)_6_ L_R_, in which a rod-linker polypeptide (L_R_) is bound to the central hole of the two stacking trimers. Unlike the B-PE and R-PE subunits, the L_R_ of C-PE-I does not carry phycobilins [31]. C-PE-II, found in marine cyanobacteria, such as *Synechocystis* sp. and *Synechococcus* sp., has five PEB chromophores in the same position as C-PE-I and a PUB molecule attached to α-75 cysteine [21]. Phycoerythrocyanin, an alternate form of PE, may be found in some cyanobacteria [16]. It exists in trimeric (αβ)_3_ or hexameric (αβ)_6_ forms, each containing a PVB chromophore at the α-84 residue and two PCB linked to the β-84 and β-153 residues [16,21]. When some cyanobacteria acclimate to light intensity and quality variations, the chromophore composition of C-PE can be modified through the CCA process [32]. PE has a quantum yield of up to 0.98 and a molar coefficient of up to 2.4 × 10^6^. Since it has superior spectral characteristics, PE outperforms the commonly used fluorescent dyes (fluorescein and rhodamine) and is used as a fluorescent probe [13].

## 3. C-PE Producer Species

**Figure 3:**
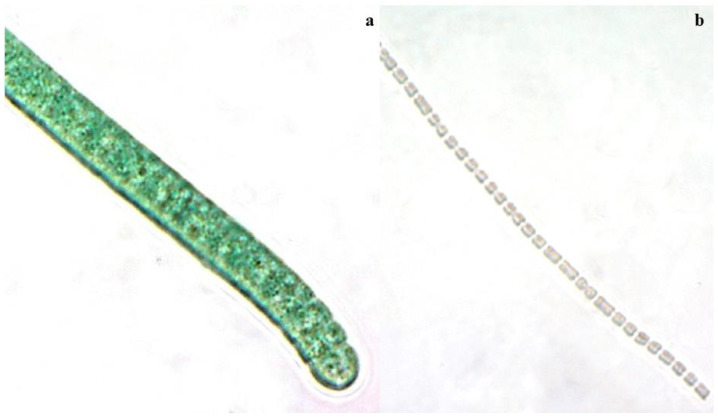
Potential cyanobacteria strains that produce C-phycoerythrin: (**a**) *Tolypothrix* sp.; (**b**) *Pseudanabaena* sp.

The current commercial PE is derived from red algae, either microalgae or macroalgae. Such production is governed by their species and metabolism. In different species, the R-PE content might range from 0.14 mg g^−1^ to 54.3 mg g^−1^ [34,35]. *Palmaria palmata* has the highest content among the studies [34]. R-PE production is, therefore, considered insufficient to satisfy global market demand. Aside from that, red algae such as *Porphyridium*, *Ceramium*, and *Porphyra*, contained a large amount of polysaccharide, which formed a gel (carbohydrate), making the purification of PE procedures tedious and laborious [36]. The high cost of cultivation and production spurs researchers to improve the production process or look for a new source of PE. Cyanobacteria are a great source of PBP because they are fast-growing microorganisms with high PBP contents, particularly in PC content. Unlike most algae, cyanobacteria could thrive in open ponds under adverse conditions [13]. The unique features of CCA and nitrogen fixation ability have considerably reduced cultivation costs and made cyanobacterial growth easier [3,16]. To date, numerous cyanobacteria species, including *Tolypothrix* sp. (660 mg g^−1^), *Nostoc* sp. (125.11 mg g^−1^), *Anabaena* sp. (102 mg g^−1^), and *Pseudanabaena* sp. (92.57 mg g^−1^) have been reported to have higher C-PE content than red algae (Figure 3) [37,38,39,40]. These species have been extensively studied for C-PE synthesis and optimized for improved productivity in the literature.

On the other hand, the biosynthesis of PE in a heterologous host is an efficient method for producing recombinant PE. The co-expression of multiple genes for chromophore and apoprotein synthesis, as well as chromophore attachment, is essential for the production of recombinant PBP [41]. Recombinant PBP has been explored since 1980 and is mostly expressed in *Escherichia coli*. However, there are still some limitations (i.e., the inefficiency of chromophorylation) in recombinant PBP that need to be resolved before it can be utilized commercially. Chen et al. (2022) have summarized the characteristics and research literature on recombinant PBP. The vast advancements in genetic manipulation technologies, such as the CRISPR (clustered regularly interspaced short palindromic repeats), make the development of a stable PBP pathway in the host more feasible and would enhance PBP production [13]. 

## 4. Cultivation Methods

Cyanobacteria can be cultivated in open systems (tanks/ponds or raceways) or closed systems (photobioreactors). The high risk of contamination is a critical issue that impedes the mass production of C-PE in open systems [3]. Enclosed photobioreactors are recommended as a preferable choice for pure, large-scale C-PE production since they allow for consistent light provision, temperature control, and carbon dioxide distribution as well as reduced contamination risk [41]. 

### 4.1. Cultivation Mode

Cyanobacteria cultivation can be carried out in a variety of modes, including heterotrophic, photoautotrophic, and mixotrophic settings. Heterotrophic mode is usually performed in dark or harsh environments where cyanobacteria need to acclimate to the environment by consuming available organic compounds (i.e., acetate, glucose) as a carbon source [42]. Although the organic compound promoted the growth of cyanobacteria, it also increased the potential for contamination, resulting in health risks within food industries [43]. Photoautotrophic cyanobacteria use natural sunlight as an energy source and atmospheric air as a carbon dioxide supply. As a result, when the light intensity is too high (photo-inhibition) or too low (photo-limitation), the biomass is affected and reduced [3]. Mixotrophic cultivation combines heterotrophic and photoautotrophic modes; both are performed in the presence of sunlight and carbon sources. Specifically, it is a dual-limiting system in which low light intensities or low organic carbon substrate concentrations can limit cell growth. At the same time, high light intensities or high carbon substrate concentrations can also suppress cell growth [43]. Previous research has shown that cyanobacteria cultivated in a mixotrophic environment are more resistant to light stress than photoautotrophs [44]. However, not all cyanobacteria can grow in mixotrophic or heterotrophic conditions [3]. 

### 4.2. Light 

Light condition is the major factor that affects C-PE accumulation. This condition involves not just light intensity but also light color and photoperiod. It has generally been documented that there is a preference for low to medium light intensity for C-PE production [16]. This is related to the role of PE in increasing the range of light absorption in the photosynthetic system. Thus, in low light-intensity conditions, the cyanobacteria will stimulate PE production to capture more light and energy for growth.

In terms of light color, previous research has found that cyanobacteria cultivated under green light accumulate more C-PE compared to other light spectra (Table 1) [38,45,46]. Under green light, the PSII overexcites and induces a transition to State II, resulting in more excitation energy transfer from the PSII to the PSI. This transfer enables PE to get the green light and use it to activate the photochemistry of both photosystems (PSI and PSII) [47]. Hence, the light color changed the composition of phycobilisomes via CCA. Hsieh Lo et al. (2019) have discussed the mechanism of CCA, but further study is needed to explore it in detail [3]. Further, Mishra et al. (2012) postulated that C-PE production could be stimulated under blue light since the light absorption PE ranges from 450 to 570 nm, allowing it to capture more blue light (460–475 mm) [46,48]. On the other hand, photoperiod affects the balance of heterotrophic and photoautotrophic metabolisms. Although scientific literature has shown that a dark period is always required for phycobiliprotein production, the optimal photoperiod for C-PE accumulation has yet to be determined [49,50]. Literature has shown that a photoperiod of 16 hours of light and 8 hours of darkness was the ideal condition for PBP synthesis [16,50]. Nevertheless, most research on C-PE yield used a photoperiod of 12 hours of light and 12 hours of darkness in their study, despite the fact that this parameter has not been optimized for C-PE yet [4,38,40,46,51].

### 4.3. Temperature, pH, and Salinity

Temperature is another major factor influencing cyanobacterial growth and C-PE productivity since it directly impacts cell membrane fluidity, nutrient availability, and absorption [3,49]. The ideal temperature for target pigment synthesis varies with different cyanobacterial species or strains, depending on their adaptability and tolerance [3]. Extremely high temperatures denature enzymes or proteins, inhibiting growth and decreasing C-PE production, whereas extremely low temperatures will cause certain cyanobacterial metabolism to malfunction [54]. Previous studies reported that temperature in the range of 30–36 °C was ideal for the growth of most cyanobacteria [49]. For example, the optimal temperature of *Anabaena* sp. was reported at 30 °C (Table 1) [38]. Studies to optimize temperature, focusing on C-PE production, still received little attention.

pH fluctuations affect nutrient solubility and bioavailability, the transportation of molecules across cytoplasmic membranes, enzyme activity, photosynthetic electron transport, and cell physiology [39,53]. Most cyanobacteria have been found to prefer an alkaline environment for growth and phycobiliprotein production. Extreme pH levels will change the charge on protein, eventually leading to protein denaturation [39]. The ideal pH for cyanobacteria growth is strain-dependent, ranging from 6.0 to 10.0 and never below a pH of 3.0 or exceeding a pH of 12.0 [16]. However, the study of the optimal pH for high C-PE production is still limited to a few strains (Table 1). The neutral pH of 7.0 is reported to be ideal for high C-PE accumulation in *Nostoc* sp. and *Spirulina platensis*, while a pH of 8 is suitable for *Anabaena* sp. and *Nodularia sphaerocarpa* [39,48,52,53].

Salt stress decreases plant growth and metabolite production, which are usually related to lower photosynthesis [55]. The optimal salinity depends on the strain. Halophilic cyanobacteria can thrive in hypersaline environments, but high salinity can inhibit the photosystem and electron transport chain in non-tolerant strains [53,56]. Furthermore, excessive sodium ions can cause phycobilisome detachment from the thylakoid membrane, reducing photosynthetic activity and nutrient absorption [49,57]. Pagels et al. (2019) compiled a few pieces of research that looked into the effect of salinity on the growth and phycocyanin production of marine cyanobacteria [16]. Yet, there is a lack of research on the effect of salinity on C-PE production (Table 1). Sharma et al. (2014) optimized the salinity level suitable for *Spirulina platensis* and found that a 0.4 M salt concentration is optimal for increasing the C-PE content [53]. 

### 4.4. Nutrients (Nitrogen, Carbon, and Phosphorus)

Nutrient uptake is crucial for cyanobacterial growth and pigment production. Nitrogen is one of the important macronutrients since it is required to synthesize nucleic acids and proteins [58]. Furthermore, nitrogen, which serves as the primary nitrogen store within the cell, is essential for cell viability and can regulate the production of PBP [59]. The nitrogen supply in the growth medium influences metabolite production, as it alters the nutrient assimilation mechanism within the cell [16]. Nitrogen deprivation can impair cellular development rates and contribute to a phenomenon known as chlorosis, which provokes PBP degradation and leads to photosynthesis downregulation [59,60]. The type of nitrogen source and the amounts required are species-dependent [16]. Cyanobacteria have been demonstrated in studies to be capable of assimilating a wide range of nitrogen sources, including nitrate (NO_3_^−^), ammonium (NH_4_^+^), nitrite (NO_2_^−^), and urea (Table 2). A few research studies have compared various nitrogen sources on C-PE synthesis. Most cyanobacteria (including *Arthrospira platensis*, *Fischerella* sp., *Nodularia sphaerocarpa*, and *Spirulina maxima*) prefer nitrate concentrations ranging from 0.2 to 2.5 g L^−1^ [52,61,62,63]. *Anabaena fertilissima* supplemented with nitrite reported the best C-PE production, whereas an ammonium source was reflected in *Phormidium* sp. and *Pseudoscillatoria* sp. [48,61]. The addition of an inappropriate nitrogen source (especially ammonium) will result in a significant influx of ammonium ions and a change in pH, which will be toxic to the cell and reduce C-PE production [16,61]. Liotenberg et al. (1996), on the other hand, discovered that ammonium and nitrate sources may alter PBP proportion differentially depending on the cyanobacterial strain. The study revealed that the growth of *Calothrix* sp. in the presence of ammonium, as compared to nitrate, increased the PC content by 46% but resulted in 35% lower intracellular levels of PE [64]. Given that a PE monomer contains more tetrapyrrole chromophores than the other two phycobilin monomers, it may be assumed that fewer chromophores are required to construct phycobilisomes in ammonium-grown cells than in nitrate-grown cells [64,65]. As phycobilisomes constitute a main cellular investment, with phycobiliproteins accounting for up to 50% of the cell’s soluble protein, a lower nitrogen level, per tetrapyrrole, might be more suitable in nutrient-limited conditions [66]. This statement was consistent with the findings of Hemlata (2009) and Simeunovie et al. (2013) that *Anabaena* sp. and *Nostoc* sp. produced higher C-PE when no additional nitrogen source was supplied [49,67]. Further, the nitrogen fixation capabilities of certain cyanobacteria (i.e., *Nostoc* sp., *Oscillatoria* sp., and *Trichodesmium* sp.) can be driven when the inorganic nitrogen source is limited and inhibited when the inorganic nitrogen source is abundant [53,68]. Non-nitrogen-fixing cyanobacteria are normally limited by nitrogen, but nitrogen-fixing cyanobacteria may use ubiquitous atmospheric nitrogen, giving them a competitive advantage in nitrogen-limiting conditions [69]. Yet, although some cyanobacteria can fix atmospheric nitrogen, it is typically preferable to supply the medium with nitrogen [16]. 

Phosphorus has major structural roles in nucleic acids and is functional in various metabolic processes [59]. A phosphorus deficiency will affect respiration, photosynthesis, and the activities of ATP-dependent enzymes [59,70]. However, phosphorus itself has little effect on growth and pigment content [58]. Thus, only a few studies on the correlation between phosphorus and C-PE productivity have been published, and the main phosphorus source comes from K_2_HPO_4_ [62,71]. Instead, the effect of the nitrogen:phosphorus (N:P) ratio has received great attention [59,72,73]. N_2_-fixation is phosphorus-dependent, as the process needs a high amount of photosynthetically derived energy [74]. Hence, an optimal N:P ratio is crucial. While the N:P ratio can reveal which of these two nutrients is the underlying limiting factor of cyanobacterial growth and pigment synthesis, it is also a better indicator than each nutrient’s absolute concentration [72]. The extreme nutrient-limiting condition is usually encountered when the external N:P is less than 20:1 [73].

Carbon is another essential nutrient that contributes to all organic compounds, and a sufficient carbon source enables cyanobacteria to proliferate and accumulate metabolites of interest [3]. Carbon, like nitrogen, can be utilized by cyanobacteria in both organic and inorganic sources. The flexibility of shifting toward different sources makes the mixotrophic cyanobacteria more independent of light, which does not limit growth as in autotrophic mode, and carbon assimilation can be supplemented by organic compounds, as in the heterotrophic mode. To be more specific, mixotrophic cultured cyanobacteria can use resources more efficiently and produce more biomass by taking advantage of both heterotrophic and photoautotrophic conditions. The higher biomass may cause cells to self-shade, boosting pigment synthesis, such as PE [58]. The dose of carbon sources varied between strains and was highly dependent on light conditions and culture period [16]. For example, upon pretreatment with either light or darkness, *Westiellopsis prolifica* used exogenous organic carbon more quickly in the light than in the dark incubation [75]. On the contrary, *Calothrix elenkenii* utilizes glucose more quickly in the dark, especially after prolonged incubation [76]. The culture media of cyanobacteria typically contained NaHCO_3_ as the carbon source [53,71,77]. Most research concluded that sucrose was the best organic carbon source. Sucrose (5 g L^−1^)^−1^ has been shown to enhance C-PE concentration in *Anabaena azollae*, *Anabaena fertilissima*, and *Nodularia sphaerocarpa* (up to 90%) when compared to fructose and glucose [48,52,78]. Sugarcane molasses, as an alternate source of pure sucrose, has been found to be the most promising substrate for producing C-PE in *Nostoc* sp. [79]. PBP synthesis may increase in the presence of sucrose due to the production of ATP and higher energy-related assimilation [76]. The addition of glucose in the medium has also been shown to enhance growth and C-PE synthesis in *Calothrix* sp. and *Nostoc* sp. [76,79,80,81]. Furthermore, glycerol can substitute glucose in enhancing PBP synthesis in *Nostoc* sp. [80] Lignite, a low-rank carbon byproduct of numerous carbon extractions, has an organic nature and benefits microbial nutrition [16]. Hence, its use at a low concentration (0.06 g L^−1^) positively affected growth and C-PE synthesis in *Spirulina platensis* [82].

**Table 2:** The nutrient sources utilized by cyanobacteria to increase C-PE production. C-PE content is expressed in mg g^−1^ unless another unit is indicated. The asterisks (*) indicate the value is absorbance value.

Nutrients	Cyanobacteria	Source	Concentration Range (g L^−1^)	Optimal Condition (Source; Concentration (g L^−1^))	C-PE Content	Reference
Nitrogen	*Anabaena fertilissima* (PUPCCC 410.5)	KNO_3_,KNO_2_	0.2–0.5	KNO_2_; 0.2	193.2	[48]
	*Anabaena* sp.	NaNO_3_, urea, NH_4_^+^	0–0.5	0	TPB: 127.5	[49]
	*Arthrospira platensis*	NaNO_3_, KNO_3_, NH_4_Cl	2.4	NaNO_3_; 2.4	1.84%_DW_	[61]
	*Fischerella* sp.	NaNO_3_, NH_4_^+^	0–1.0	NaNO_3_; 0.2	126.5	[63]
	*Nodularia sphaerocarpa* (PUPCCC 420.1)	KNO_3_, NaNO_2_	0.2, 0.5, 1.0, 1.5	KNO_3_; 0.5, NaNO_2_; 1.0	n/a (Increase 40%)	[52]
	*Nostoc* sp. (S36)	NaNO_3_	0, 2	0	~ 160	[67]
	*Phormidium* sp.	NaNO_3_, KNO_3_, NH_4_Cl	1.5	NH_4_Cl; 1.5	1.67%_DW_	[61]
	*Pseudoscillatoria* sp.	NaNO_3_, KNO_3_, NH_4_Cl	1.5	NH_4_Cl; 1.5	1.36%_DW_	[61]
	*Spirulina maxima*	NaNO_3_	2.5	NaNO_3_, 2.5	0.02	[62]
						
Phosphorus	*Oscillatoria* sp. (OSCI_UFPS001)	K_2_HPO_4_	0.04	K_2_HPO_4_; 0.04	1.8%_DW_	[71]
	*Spirulina maxima*	K_2_HPO_4_	0.5	K_2_HPO_4_, 0.5	0.02	[62]
						
Carbon	*Anabaena azollae*	Glucose, jaggery, sucrose	5	Sucrose; 5	0.2 (Increase up to 90%)	[78]
	*Anabaena fertilissima* (PUPCCC 410.5)	Fructose glucose, sucrose	5	Sucrose; 5	197.9	[48]
	*Calothrix elenkenii*	Glucose	5	Glucose; 5	0.12	[76]
	*Calothrix* sp.	Glucose	1	Glucose; 1	n/a	[81]
	*Nodularia sphaerocarpa* (PUPCCC 420.1)	Fructose glucose, sucrose	5, 10	Sucrose; 5	n/a (Increase 40%)	[52]
	*Nostoc* sp.	Glucose, sugarcane molasses, sucrose,	0-5	Glucose; 1	2.633 *	[79]
				Sucrose; 0.5	0.84 *	
				Sugarcane molasses; 1	1.44 *	
	*Nostoc* sp. (2S7B)	Glucose, glycerol	0–3	Glycerol; 3	n/a	[80]
	*Oscillatoria* sp. (OSCI_UFPS001)	NaHCO_3_	0.16	NaHCO_3_; 0.16	1.8%_DW_	[71]
	*Spirulina platensis*	NaHCO_3_	0, 4.5, 9, 18	NaHCO_3_; 9	1.97	[53]
		Lignite	0–0.06	Lignite; 0.06	0.60	[82]
	*Trichromus* sp.	NaHCO_3_	0.02	NaHCO_3_; 0.02	5%_DW_	[77]

n/a represents not available; DW represents the dry weight.

## 5. Downstream Processing of C-PE

### 5.1. Extraction of C-PE

C-PE downstream processes are typically multi-step and sophisticated in order to assure quantity, purity, and quality [4]. A proper extraction should allow for the recovery of the greatest amount of C-PE with minimal contamination, which will be valuable for future analyses of its chemical structure and biological activity [4,83]. 

The selection of the solvent is primordial for efficient C-PE extraction. The choice of extraction buffer must take into account not merely the polarity and solubility of C-PE, but also the economics, availability of the solvent, and simplicity of use [4,16]. The phosphate buffer and tris chloride buffer are the common buffer types reported for the extraction of C-PE (Table 3). These buffers have been widely used for PBP extraction in cyanobacteria and seaweed and have been proven effective [24,84,85]. An acetate buffer at a pH of 5 is occasionally employed as a buffer for C-PE extraction [86]. However, research by Julianti et al. (2019) and Zavrel et al. (2018) demonstrated that the C-PC yield and purity extracted with an acetate buffer are lower than that extracted with a phosphate buffer [87,88]. A comparatively low-cost, double-distilled water was shown to extract the highest quantity of C-PC, with no significant difference in comparison to PC amounts extracted using a phosphate buffer [89]. However, there is no information on utilizing double-distilled water for C-PE extraction. The pH solvent used in most C-PE extractions was between a pH of 7 and a pH of 8. (Table 3). Dilute or low-molarity buffers could provide better extraction than high-molarity buffers due to the salting-in effect [4,90]. Since a pH of 8 is not suitable for other proteins, it can avoid undesirable contamination, making crude extract purification easier [4].

Cyanobacteria cell walls are multi-layered and difficult to disrupt, yet cell rupture is needed to extract the C-PE. The crude extraction can be accomplished using several methods, including enzymatic digestion, high-pressure homogenization, ultrasonication, or continuous freezing and thawing of the biomass [89]. The methods chosen must consider factors such as the physical strength of the cell wall, stability, and the composition of the cyanobacteria [91]. Enzymatic extraction can be completed by using lysozyme to enzymatically digest the biomass. This enzymatic hydrolysis is particularly effective in seaweed, which has a strong cell covering or outer sheath to protect the cells [92]. Nevertheless, as cyanobacterial cells lack special protection, purified enzymes may not be required. Mechanical methods, such as grinding, crushing, homogenization, or sonication, will generate heat transferred to the biomass solvent, causing the C-PE extracts to be denatured even before they are entirely extracted into the buffer [4,24]. Instead, repeated freezing and thawing were recommended, as it is the gentler approach from the protein perspective and no heat was generated throughout the process [4]. Thus, it is the most commonly used technique for C-PE extraction [36,51,84]. Ghosh and Mishra (2020) also concluded that the freezing–thawing method was the best way to achieve the cell disruption of marine cyanobacteria during their screening of several potential C-PE extraction procedures [4].

### 5.2. Purification of C-PE

Refined purification can increase the commercial value of C-PE extract. Purifying C-PE is time-consuming and labor-intensive since most cyanobacteria produce PC and APC along with the target PE [93]. Although the ultrafiltration process improves C-PE purity, it reduces C-PE concentration due to thermal and mechanical shear forces [36]. Nowadays, PBP is usually purified from crude extract using at least one of the following methods: precipitation, centrifugation, or chromatography (Table 3). According to Kamble et al. (2018), combining different methodologies is more beneficial for increasing the purity of C-PE while retaining a better yield [36,86]. The first step can be completed by ammonium sulfate precipitation [16]. Ammonium sulfate is water soluble at low temperatures, which inhibits bacterial growth and assists in protein concentration and purification [94]. Different amounts and concentrations of ammonium sulfate were utilized as a salting-out agent to exclude unwanted proteins and boost the purity of C-PE [36]. The precipitated C-PE was further purified based on size, color, and polarity using chromatography approaches, including size-exclusion and ion-exchange chromatography. Size-exclusion chromatography, also known as gel-permeation chromatography, commonly uses Sephadex as a gel filtration resin to remove high-molecular-weight proteins [84,85]. Diethylaminoethyl cellulose (DEAE-C), a positively charged resin, was employed in ion-exchange chromatography to eliminate unwanted protein [36,85,86]. A more developed, aqueous, two-phase system has been used effectively as a low-cost alternative to recover C-PC, but no studies have been conducted to assess its efficiency on C-PE [95].

## 6. Strategies to Improve the Stability of C-PE

Another prerequisite for large-scale production and acceptability in the industry is the stability of C-PE [3]. C-PE is unstable and susceptible from the time it is extracted from the biomass. As C-PE is a protein, pH, temperature, or light changes may modify its molecular structure. For these reasons, C-PE degradation is possible and can be significant during or after the extraction and purification process, resulting in its shorter shelf life [96]. The pH level is the major factor influencing C-PE aggregation and dissociation, either in monomer, trimer, or hexamer forms in the solution. The most stable structure of C-PE, the hexameric form, predominates at a pH of 7 and easily dissociates at a higher or lower pH [97,98]. C-PE extracted in the study completed by Ghosh et al. (2020) was stable within the pH range of 3–8. This broad pH range facilitates its use in the food and beverage industries. As most beverages have an acidic pH, a stable colorant under such conditions is beneficial [4]. Further, it is recommended to keep extracted C-PE at a low temperature. Temperatures exceeding 40 °C reduce the number of alpha helices, resulting in a loss of stability and gradual degradation [99]. It is also not suggested to keep C-PE at temperatures above room temperature since C-PE is more sensitive to deterioration by microorganisms [97]. Sub-zero temperatures are favorable for long-term preservation since they restrict protein water activity to a minimum level [4]. Furthermore, it is preferable to keep C-PE in the dark [3]. Long-term exposure to high light intensity causes C-PE to lose its chromophores, resulting in color degradation and stability loss [99].

Several stabilizing approaches or strategies have been introduced to increase C-PE stability, as outlined by Hsieh-Lo et al. (2019) [3]. The addition of effective preservatives is the most popular and convenient method. Although dithiothreitol (DTT) and sodium azide (NaN_3_) are commonly employed in C-PE for analytical purposes, both are poisonous and not suitable for use in food [98]. Thus, only edible preservatives can be used to produce food-grade C-PE. According to Mishra et al. (2010), the stability of C-PE treated with citric acid is higher than that treated with sucrose, calcium chloride, or sodium chloride as preservatives [98]. Benzoic acid is another useful edible additive. It has potent antioxidant properties that can preserve and enhance the stability of C-PE while also acting as an antibacterial agent that inhibits bacterial growth [96].

Crosslinking is another non-additive technology used effectively on PE to increase stability [100]. It is a method of reinforcing the folded structure of a protein by covalently joining two chemical groups on its surface using bifunctional reagents, either intermolecularly or intramolecularly [101]. The intramolecular crosslinking of the protein molecule with silver nanoparticles, Ag^+^, has been shown to prevent protein aggregation and enhance the thermal stability of PE [100]. Other approaches, such as complex formation and microencapsulation, have been established on PC; however, more study is required to determine the efficacy of employing such techniques on PE [102,103,104]. At present, most of the established strategies have focused on enhancing thermal stability. Hence, stability regarding other factors needs to be explored so that extracted C-PE may be widely marketed across various fields.

## 7. Application of C-PE

### 7.1. PE in Pharmaceuticals, Nutraceuticals, & Therapeutics

Several bioactive compounds, such as zeaxanthin, α-tocopherol, and caffeic acid, have already been employed in disease prevention and treatment. However, cyanobacterial bilin is more effective than other phytochemicals, attracting the attention of researchers [105]. The study of PBP bioactivities has emerged in recent years, albeit such study is still restricted and mainly focused on PC [16]. Indeed, PE displays a variety of bioactivities that allow it to be used in the nutraceutical, pharmaceutical, and therapeutic industries.

The accumulation of reactive oxygen species (ROS) causes oxidative stress, which stimulates organisms to produce bioactive compounds in response to their defensive mechanisms. An enzymatic or non-enzymatic antioxidant approach can neutralize these ROS. PBP is an example of a non-enzymatic antioxidant mechanism that scavenges ROS and reduces oxidation [16]. Sonani et al. (2014) discovered that C-PE isolated from *Lyngbya* sp. exhibited higher in vitro, dose-dependent, antioxidant activity than C-PC and C-APC [85]. This might be because C-PE exhibits antioxidant activity through the main pathway, scavenging already produced ROS via a redox reaction, and has lower chelating and reducing ability [106]. Other potential antioxidant properties of C-PE still require further investigation. In addition to its antioxidant capabilities, C-PE from *Lyngbya* sp. also had anti-Alzheimer’s and anti-aging properties [85,106]. Phycobilins, such as PCB, PEB, PUB, and PVB, have also been potent phytochemical inhibitors of SARS-CoV-2 Mpro and PLpro proteases [107].

Anti-tumor activities and underlying mechanisms were primarily proven in the literature employing C-PC in medications [16,108]. C-PE differs structurally and spectroscopically from C-PC, suggesting that it may potentially have unique anti-tumor properties [13]. R-PE has been incorporated into photodynamic therapy (PDT), a treatment method for lung, stomach, skin, and oral tumors [13,109]. Effective PDT can selectively destroy cancer or tumor cells by producing ROS-mediated damage, vascular damage, and immune system activation while causing no damage to normal cells [13]. R-PE and its subunit had a stronger PDT effect as well as an inhibitory effect on human liver cancer cell SMC 7221 and mouse tumor cell S180. The smaller size of the β-subunit allows it to access the tumor cell easily. Furthermore, it emitted more significant fluorescence, which was employed as a fluorescent marker for detecting binding sites [109]. In this regard, future research could focus on the role and dosage of C-PE in tumor therapy.

Oral treatment with C-PE as a hepatoprotective and neuroprotective drug has ameliorated kidney, redox, hepatobiliary, and hepatocellular biomarkers against CCl_4_-induced toxicity in rats. It was determined that proteolytic enzymes might break down C-PE in the gastrointestinal system into bilirubin and low-molecular-weight proteins, mediating the pharmacological effects [110]. Furthermore, C-PE was shown to alleviate diabetes complications by lowering oxidative stress and oxidized, low-density, lipoprotein-induced atherogenesis in streptozotocin-induced type 2 diabetic mice. C-PE administration decreased organ weights, food consumption, cholesterol concentrations, and serum concentrations of glucose and raised body weight, bilirubin, total protein, and the ferric-reducing capacity of plasma values [111]. Also, hepatic and renal tissues showed substantial reductions in lipid hydroperoxide, thiobarbituric acid reactive substances (TBARS), and conjugated diene contents, with elevations in superoxide dismutase, catalase, and glutathione peroxidase, and reduced glutathione, vitamin C, and vitamin E levels [13,111]. Although C-PE administration has become widely used, the detailed mode of action of C-PE in many diseases remains unknown. Further study is needed to elucidate the action point of C-PE in different metabolic pathways.

### 7.2. PE in Cosmetics

C-PE has long been recognized as an excellent quencher of various oxygen derivatives. Due to this capability, phytopigment of *Spirulina* sp., such as C-PC, PCB, and PEB, are thought to be excellent antioxidant, anti-wrinkle, anti-melanogenic, and anti-aging agents, as well as natural, non-toxic colorants in eye shadows, eyeliners, and lipsticks [14,112,113]. The majority of the PE content in commercial cosmetics and skin care products is in the form of B-PE or R-PE; however, there is no data on PE derived from cyanobacteria [14,15,114]. For example, purified R-PE isolated from *Colaconema formosanum* demonstrates anti-aging and anti-allergic properties without toxicity on several mammalian cell lines and epidermal tissues, indicating that this compound has potential for cosmetics usage [15]. Various cyanobacteria strains are now commonly utilized in skin care products to treat various skin problems by functioning as anti-wrinkling agents, sunscreens, nourishing moisturizers, whitening agents, or texture enhancers [113,115]. Hence, it is envisaged that C-PE, which has a range of beneficial bioactivities, will be included in cosmetics or skincare products.

### 7.3. PE in Food and Feed Industries

By appealing to consumers’ growing health awareness, PE with natural colorant properties has become an alternative to the use of synthetic dye [41]. For example, the pink colorant exhibited by PE has been added as a dye into dairy products, such as milkshakes and yogurt, to make processed products more enticing and to provide color to otherwise colorless food [116]. Red phycoerythrin has a unique yellow fluorescence. Transparent lollipops prepared from sugar solution, soft drinks, dried sugar-drop sweets for cake decorating (that fluoresce under UV light), and fluorescent alcoholic beverages that exploit the benefits of these spectral properties are still being researched [117]. Another pivotal reason for using PE as a dye is that it has antioxidant properties that add value to processed food products. Phycoerythrin is famous as an antioxidant and anti-inflammatory drug, with features that are able to avert ROS-related abnormalities, protect against physiological changes caused by oxidative stress, and have anti-aging benefits [16,118]. This substance also has fewer side effects than synthetic additives or chemical drugs. However, C-PE uses in food products need to be explored further and corroborated through toxicity testing. Further, more study on the bioavailability and interaction of diets and C-PE is necessary [116]. 

Several studies have demonstrated the benefits of using PE as feed. For example, Lee et al. (2021) found that PE can variably increase the immunological response of whiteleg shrimp in vitro and in vivo and that it might be applied as an immunomodulator in shrimp production [119]. Dietary PE supplementation may also modify the gut microbiota to improve intestinal nutrition and disease resistance in animals by increasing the beneficial bacteria and reducing the prevalence of harmful bacteria within the intestine [120].

### 7.4. PE in Detection, Diagnosis, and Biotechnology

Compared to monomers, the high molar extinction coefficient of PE is ascribed to trimeric and hexameric packing, as well as the presence of open-chain tetrapyrrole chromophores covalently attached to them [41,119]. The unique fluorescent features of PE have broadened its applications as a fluorescent label in immunoassays, flow cytometry, cell biology, and fluorescence microscopy for biomedical research and diagnostics [121,122,123]. Furthermore, the staining of the PE-labelled monoclonal antibody probe was more clear and bright than the PC-labelled probe when detecting invasive amebiasis [124]. PE can also be employed as a marker in electrofocusing or gel electrophoresis [125] and as a photosensitizer in cancer therapy [126]. 

## 8. SWOT Analysis of C-PE Derived from Cyanobacteria

**Figure 4:**
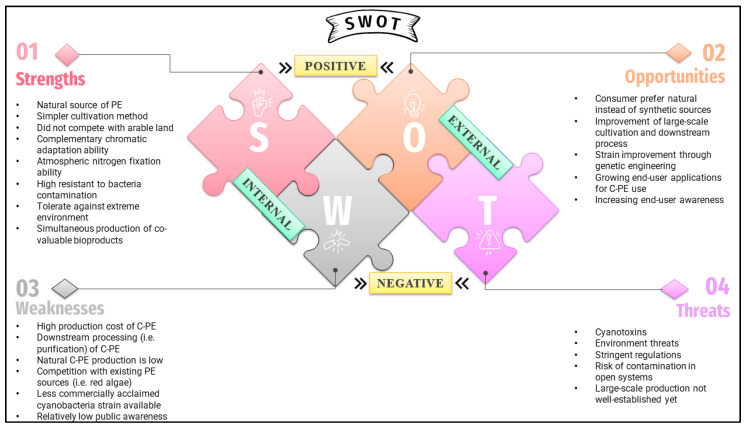
Strengths, weaknesses, opportunities, and threats (SWOT) analysis of C-PE.

The SWOT analysis is employed to assess the importance of the four main components (strengths, weaknesses, opportunities, and threats) in achieving the economic viability of cyanobacteria-derived C-PE (Figure 4). This analysis appraises the current state and forecasts of the worldwide C-PE markets.

### 8.1. Strengths and Opportunities

C-PE is being pursued due to its natural origin. Cyanobacteria have attracted more interest than eukaryotic microalgae as the sources of bioactive compounds due to their easier culture methods and higher resistance to bacterial contamination [25]. Cyanobacteria are capable of surviving in a wide range of adverse conditions. Their living processes rely on the bare necessities of light, carbon dioxide, water, and inorganic compounds [127]. Furthermore, cyanobacteria may be produced without arable land and they might be the first plants to colonize bare rock and soil habitats [127]. In microalgae, phycobiliprotein, which functions as a nitrogen reserve, will break down under nitrogen deprivation. According to Da Silva et al., the nitrogen-starved cell of *Rhodomonas* causes a 25-fold reduction in PE content [128]. In contrast, cyanobacteria can satisfy their own nitrogen requirements through nitrogen fixation [129,130]. This peculiarity reduces the cost of the culture media while reducing the problem of contamination by other microorganisms [38]. Since cyanobacteria own a chromatic adaptation mechanism, green light can be applied to enhance phycoerythrin biosynthesis [16]. The co-production of other valuable compounds, such as vitamins and proteins, also corresponds to additional value [131].

Future research potential includes exploring emerging rare C-PE sources and more profound studies of C-PE and its novel technologies and applications. To date, only a minority of cyanobacteria (i.e., *Arthrospira* sp., *Nostoc* sp., and *Oscillatoria* sp.) are well characterized and commercially exploited. The lesser-known potentially PE-producing strains, such as *Pseudanabaena* sp., can be explored further [36,132]. The expanding applications of C-PE and consumer acceptance of C-PE will position the industry for revenue growth. The development of high-value markets (both niche and commodity) will also drive research. Moreover, the prokaryotic nature of cyanobacteria allows for more superficial genetic manipulation, resulting in hyper-producing strains [133]. There is also a vast market opportunity in developing improved large-scale culture systems (i.e., effective cyanobacterial screening and strain optimization) and economic, efficient, and environmentally friendly C-PE downstream processing techniques with a focus on the circular economy and sustainability. The end-user is expected to shift with a growing interest in lifestyles of health and sustainability (LOHAS) that prioritize the production of natural C-PE from sustainable sources [134].

### 8.2. Weaknesses and Threats

Natural C-PE production is problematic because it is labor-intensive and costly [14]. The production cost of high-purity PE was around 30 USD per gram [135]. The high production costs limit the commercial value of C-PE and reduce its market share. C-PE-producing cyanobacteria are often reported to exhibit a slower growth rate and low PE production, along with being recalcitrant, making C-PE extraction and purification even more laborious [3]. Low PE content in cyanobacteria can be rectified using cost-effective process optimization methods [3,16]. A high yield of C-PE can also be achieved through strain improvement via genetic engineering [13]. The downstream processes for extracting C-PE from cultures have become significant challenges since they must adhere to as many green chemical principles as possible to last in the future [58]. Several methods, including maceration, ultrasound, microwaving, freeze–thaw extraction, pressurized liquid/solvent extraction, high-pressure homogenization, and sub and supercritical fluid approaches, have been tested in recent years to extract PE from various sources [4,24]. These are intriguing strategies, and more research will be conducted in the coming years to confirm the findings and enhance pigment selectivity. Conventional solvent extraction methods, such as phosphate-buffered saline and sodium or potassium phosphate buffers are the most well-documented. Nowadays, these solvents are generally replaced with double distilled water, a more environmentally friendly solvent with outstanding results and a high extraction yield [89]. Following extraction, another difficult task is purification. Unlike cryptophytes, cyanobacteria produce phycobilisome protein complexes that usually contain allophycocyanin and phycocyanin. This complicates protein purification in C-PE production since distinct PBPs need to be separated from one another [93]. The chromatography approach appears to be the most promising purifying method, as it is based on a solvent system similar to green solvents used for extraction [36]. There are currently fewer commercially acclaimed cyanobacteria strains available. The rivalry from currently existing natural commercial sources of PE, such as rhodophyte, poses hurdles to the acceptance of cyanobacteria-derived PE [1,24].

Extensive research on cyanotoxins is required to completely comprehend their adverse effects on humans and the environment. Despite their numerous benefits, cyanobacteria can trigger algae blooms, which endanger the environment and ecosystem [136]. As a result, deep insight into the biosynthesis pathway and cyanotoxin removal methods is required to pave the way for the safe exploitation and expansion of the applications of cyanobacterial compounds [137,138]. Strict rules and slow-moving legislation may impede the path to market and the development of novel products. Different countries and geographical areas have different safety and regulatory standards. Companies that intend to market products containing microalgae (including cyanobacteria) must follow the legislation in place, which often involves submitting scientific information and health and safety assessments to a region-specific authority for approval [134]. Only a few algae, such as *Porphyridium* sp. and *Arthrospira* sp., are regarded as generally recognized as safe (GRAS) for human consumption by the Food and Drug Administration (FDA) [126]. Biotechnology for species modification (genetic engineering) must also consider health standards [139]. Aside from this, there is a lack of efficient, large-scale C-PE production. It is noteworthy that each cyanobacteria species has specific growth requirements that must be studied prior to large-scale production. Also, further research is needed to deal with the contamination issue in open cultivation systems [16].

## 9. C-PE Market Drive and Commercial Relevance

Despite a growing interest in algal resources, European production lags behind global production. This is due to the scale and competitiveness of Asian algal aquaculture and the scarcity, ambiguity, and fragmentation of information accessible in the sector [134,140]. The existing regulatory framework for the algae industry may need to be optimized to consider market data and construct a database on algal production and potential markets [140]. To commercialize C-PE worldwide, the limitations, needs, and opportunities for supporting sustainable development in the industry must be identified and resolved.

Research on C-PE proved that, as a natural ingredient, this pigment is an eco-friendly alternative to pharmaceuticals, food supplements or additives, and cosmetic compounds [1,3,16]. There is a high market demand for PE added-value products at present. Still, there is a lack of information regarding effective downstream processing and reducing production costs that is needed to spark the market’s interest. Although some cyanobacteria strains are efficacious C-PE producers, the downstream process has yet to be standardized. This offers a massive obstacle for C-PE to be recognized as an economically viable natural ingredient, as it should be produced through a rapid, simple, and cost-effective method. Nowadays, most reported, commercially available PE products are isolated from the well-established source, rhodophyte in R-PE form, rather than from cyanobacteria [3]. Concerns about cyanotoxins have hampered the acceptance of cyanobacteria-derived products [137]. The difficulties of preserving products for long periods of time have also curtailed PE usage, particularly in food products, resulting in a reduction in available commercial PE-containing products. Nonetheless, improvements in research on enhancing PE stability may allow for the development of a new market for C-PE-based products [3]. 

The potential applications of PE are determined by its purity index (A_565nm_/A_280nm_). A purity value of 0.7 is considered food grade, values between 0.7 and 3.9 are classified as reagent grade, and a value greater than 4.0 is regarded as analytical grade [141]. The market price of purified PE ranged between 180 and 250 USD per milligram, depending on the final purity level and intended applications [142,143]. When PE is extracted for food industries, purity is not a concern, and the price can be easily reduced. However, when used for scientific analysis, pharmaceutical applications, or as a fluorescent agent, PE purity must be higher, and the cost can be a hundred times higher. In fact, PE is sometimes more expensive than PC (15 USD per milligram) [16]. The market size for C-PE is challenging to estimate due to a lack of regional statistics and few technology products. However, the market price for PE is expected to reach $6.3 million by 2025 [24]. According to Future Market Insights Inc., the total market in 2022 is estimated to be worth 2.6 billion USD, with the potential to double that figure by 2032. The report also stated that Europe is anticipated to expand the PE market at a lucrative rate throughout the forecast timeline due to an increase in cosmetic product sales in Italy, France, and Germany over the next decade [144].

## 10. Conclusions

Cyanobacteria are remarkable blue-green factories that produce several immense market-value compounds despite a minority with toxin productions. However, a complete understanding of C-PE remains underexplored. The current work has shed light on the pertinent knowledge gaps regarding C-PE to increase the feasibility of commercial PE production using cyanobacteria. The interest in PE has burgeoned lately due to its plenteous health-promoting properties. C-PE exists in two forms: C-PE-I (freshwater and soil cyanobacteria) and C-PE-II (marine cyanobacteria). Cyanobacteria possess several attractive attributes, such as a short life cycle, higher biomass production, nitrogen-fixing abilities, and photosynthetic efficiency, as well as a high amount of PE, thus suggesting their ability to effectively substitute the current commercial source of PE (red algae). So far, *Tolypothrix tenuis* has displayed the highest rate of C-PE production at 660 mg g^−1^. This study revealed higher C-PE content was induced under green/blue light. In addition, repeated freezing–thawing is the prevalent approach in extracting C-PE. The stability of C-PE is one of the crucial aspects required prior to large-scale production; thus, several strategies were included in this study to improve the stability of this pigment. Furthermore, the understanding of C-PE is greatly enhanced via SWOT analysis, which underlined the strengths, opportunities, weaknesses, and threats of C-PE production. The development of holistic, integrated, up- and downstream approaches could substantially affect the feasibility of C-PE production. More intensive research should focus on nanotechnology, innovations in cultivation, methods of optimization and bioengineering techniques, the integration of synergistic extraction strategies, and economic and environmental assessments of C-PE production. 

## Figures and Tables

**Table 1:** The optimum cultivation conditions of cyanobacteria to achieve high yields of C-phycoerythrin (C-PE). Cultivation parameters include culture media (M), light intensity (I), photoperiod (LP), light color (LC), pH, temperature (T), and salinity (S). Light intensity is expressed in μmol m^−2^ s^−1^ unless another unit is indicated. Parameters highlighted in red color indicated the factors optimized in the respective study.

Cyanobacteria	Optimal Cultivation Parameters	Cultivation Mode	C-PE Content (mg g^−1^)	References
*Anabaena* sp.	**M**: BG 11 + HEPES; **I**: 100; **LP**: 12:12 h; **LC**: Green; **pH**: 8; **T**: 30 °C	autotrophic	102	[38]
*Anabaena fertilissima* (PUPCCC 410.5)	**M**: Chu-10 medium; **I**: 44.5; **LP**: 14:10 h; **LC**: white; **pH**: 8; **T**: 28 °C	mixotrophic	105.8	[48]
	**M**: Chu-10 medium; **I**: 44.5; **LP**: 14:10 h; **LC**:blue; **pH**: 8; **T**: 28 °C		214.5	
*Fremyella diplosiphon*	**M**: BG 11 + HEPES; **I**: 15; **LP**: n/a; **LC**: Green; **pH**: 8; **T**: 27 °C	autotrophic	559.69	[45]
*Gloeotrichia* sp.	**M**: BG 11 + HEPES; **I**: 15; **LP**: n/a; **LC**: Green; **pH**: 8; **T**: 27 °C	autotrophic	414.18	[45]
*Lyngbya* sp. (CCNM 2053)	**M**: ASN-III; **I**: 60; **LP**: 12:12 h; **LC**: n/a; **pH**: n/a; **T**: 25 °C; **S**: n/a	autotrophic	22.99	[4]
*Nodularia sphaerocarpa* (PUPCCC 420.1)	**M**: Chu-10 medium; **I**: 44.5; **LP**: 14:10 h; **LC**: green; **pH**: 8; **T**: 28 °C	mixotrophic	283	[52]
*Nostoc* sp. BTA-61	**M**: BG11; **I**: 54-67; **LP**: 14:10 h; **LC**: white; **pH**: 7.0; **T**: 28 °C	autotrophic	125.11	[39]
				
*Phormidium persicinum*	**M**: synthetic NM; **I**: 3000 LUX; **LP**: 12:12 h; **LC**: n/a; **pH**: n/a; **T**: 21 °C; **S**: n/a	mixotrophic	32.98	[51]
*Pseudanabaena* sp.	**M**: ASN-III; **I**: 75-110; **LP**: 12:12 h; **LC**: Green/blue; **pH**: n/a; **T**: 25 °C; **S**: n/a	mixotrophic	30	[46]
	**M**: BG 11; **I**: 40; **LP**: 12:12 h; **LC**: n/a; **pH**: 7; **T**: 25 °C	autotrophic	92.57	[37]
*Spirulina platensis*	**M**: Zarrouk’s medium; **I**: n/a; **LP**: n/a; **LC**: n/a; **pH**: n/a; **T**: 30 °C; **S**: 0.4 M	mixotrophic	1.61	[53]
	**M**: Zarrouk’s medium; **I**: n/a; **LP**: n/a; **LC**: n/a; **pH**: 7; **T**: 30 °C; **S**: n/a		1.44	
*Tolypothrix tenuis*	**M**: N-free mineral medium; **I**: 200 LUX; **LP**: 12:12 h; **LC**: Green; **pH**: n/a; **T**: 20–25 °C	mixotrophic	660	[40]

HEPES, hydroxyethyl piperazineethanesulfonic acid; ASN, artificial seawater nutrient; NM, nutrient media.

**Table 3:** The extraction and purification methods used to extract C-PE.

Cyanobacteria	Extraction Method	Solvent	Purification	C-PE Content	Purity of C-PE	References
*Halomicronema* sp. A27DM.	Freezing–thawing	Tris Cl buffer + sodium azide (3 mM), pH 8.1	Ammonium sulfate precipitation + Gel permeation chromatography (Sephadex G-150)	5.76 mg mL^−1^ (66.2%)	4.0	[84]
*Lyngbya* sp. (CCNM 2053)	Freezing–thawing	Phosphate buffer (0.1 M), pH 8	n/a	22.40 mg g^−1^	3.84	[4]
*Lyngbya* sp. (A09 DM)	Freezing–thawing	Potassium phosphate buffer (20 mm), pH 7.2	Ammonium sulfate precipitation (Triton X-100)	23.76 mg	1.59	[85]
			Ion exchange chromatography + Gel permeation chromatography (Sephadex G-150)	20.92 mg	6.75	
*Lyngbya* sp. (A09 DM)	Freezing–thawing	Tris Cl buffer + sodium azide (3 mM), pH 8.1	Ammonium sulfate precipitation + Gel permeation chromatography (Sephadex G-150)	8.89 mg mL^−1^ (64.9%)	3.7	[84]
*Phormidium* sp. (A27DM)	Freezing–thawing	Tris Cl buffer + sodium azide (3 mM), pH 8.1	Ammonium sulfate precipitation + Gel permeation chromatography (Sephadex G-150)	9.95 mg mL^−1^ (62.6%)	3.90	[84]
*Phormidium persicinum*	Freezing–thawing	Distilled water	Size exclusion chromatography	5.09 µg mL^−1^	2.39	[51]
			Ammonium sulfate precipitation	15.58 µg mL^−1^	3.99	
			Dialysis (phosphate buffer)	32.98 µg mL^−1^	4.35	
*Pseudanabaena* sp.	Sonication + Freezing–thawing	Sodium phosphate buffer (0.1 mM), pH 7.0	n/a	0.03 mg mL^−1^	n/a	[46]
*Pseudanabaena* sp.	Freezing–thawing	Potassium phosphate buffer (100 mm), pH 7.2	Ammonium sulfate precipitation	5.69 mg mL^−1^	2.10	[36]
			Ammonium sulfate precipitation + Size exclusion chromatography	5.77 mg mL^−1^	5.32	
			Ammonium sulfate precipitation + Size exclusion chromatography + Ion exchange chromatography	5.91 mg mL^−1^	6.86	
*Spirulina platensis*	Freezing–thawing	Acetate buffer [Sodium chloride (50 mM) + 0.002 M sodium azide (0.002 M)], pH 5.10	Ammonium sulfate precipitation	384 µg mL^−1^	2.59	[86]
			Ammonium sulfate precipitation + Dialysis (Acetate buffer)	299 µg mL^−1^	2.61	
			Ammonium sulfate precipitation + Ion exchange chromatography	374 µg mL^−1^	4.57	

## Data Availability

Not applicable.
